# An Accidental Proving of Balm of Gilead Buds

**Published:** 1891-02

**Authors:** W. C. Stilson

**Affiliations:** Bucksport, Maine


### AN ACCIDENTAL PROVING OF BALM OF GILEAD BUDS.

W. C. Stilson, M. D., Bucksport, Maine.

(A paper read before the Maine Homœopathic Medical Society and published in its transactions for 1890.)

As this is but one day, and there are many others to say things of benefit to us, I will give briefly an accidental proving, or a partial proving of Balm of Gilead Buds.

In my town lives Mr. E., who has quite an appetite for "something to take," and he is not so particular as some of us what he does take. His wife had a pint of rum in the house and had gathered some Balm of Gilead Buds and placed in it to use for a liniment. One evening Mr. E. was thirsty enough to take a drink of it, and in a few minutes went to bed. In a few hours he was breathing heavily, and his wife on awakening him found he could not speak, and so sent their son in great haste for me. When I arrived he was very nervous, and much excited; could not talk loud, but could speak only in hoarse whispers. Told me what he had taken, and said his throat and stomach felt very uncomfortable.

His pulse was up to 120; respiration affected; face ashy pale. He had a wild look, and often if asked a question would commence to answer but forget what he was saying in the middle of a sentence.

I hesitated what to prescribe, but thought of what my preceptor, Dr. Howe, once said to me when I asked him what he would give a child with such and such symptoms, and the reply was, "I would give him something as quickly as I could lest he get well before he got the medicine," so coffee, black and strong, was ordered.

It was with much difficulty that we could get him to take it, as he said his throat was so dry, burned, and constricted that he could not swallow. The tongue was dry, and there seemed to be no saliva in the mouth; the tonsils and uvula were red and somewhat swollen, accompanied by burning sensations. He said his throat felt as if spiders had woven webs in it. I gave the coffee often, and in a few hours there was improvement and I left him.

On my return the next day I found his condition much the same, but with less fever and not so much dryness of the mucous membrane; yet there was complete aphonia, his intellect was dull and it seemed hard work for him to think. Voices of persons in the room sounded in the distance; words spoken to him seemed as if uttered a long time before; objects in the room seemed multiplied; his head felt many times larger than its normal size; he was hungry but did not dare eat lest it should lodge in his throat and choke him; his stomach felt faint and there was belching of gas, feeling, as he said, as though he was throwing heated steam from his stomach; there was difficulty in breathing, a sensation as if he could not get a good breath; a slight, dry cough, caused by the cobwebs in his throat. The bowels had not moved, and the urine passed was of a dark straw color, and looked as if clouds of smoke were mingled with it, while surely the odor was that of Balm Gilead.

I gave medicines and the next afternoon found him much improved; could talk aloud, but still with difficulty. The bowels had moved, but the evacuations were preceded by cramps in the abdomen, the stools were small and narrow; there seemed a lack of expulsive power.

There was deficient power in deglutition, and food would stop in the œsophagus or pass with difficulty. These symptoms all grew less and subsided in a few days, and he has not had any hankering for mixed drinks since.

After this I obtained some buds and tinctured them, and in testing them have found them to prove very satisfactory in hoarse and catarrhal conditions of the throat and glottis, and in aphonia produced by catarrhal colds. This I know is of but little consequence, but when produced by causes acting upon the nervous system and without any apparent lesion of the vocal apparatus, then it becomes serious and frequently resists all treatment.

Mrs. S. had what our brother doctors called nervous prostration, and during this attack had aphonia. The nervous trouble grew better but the voice did not return. After trying several doctors without improvement, she decided to give Homœopathy a try, and I was called in. With what symptoms I could gather I decided that it was a Balm of Gilead case, and gave thirty drops in one-half glass of water, to take two teaspoonfuls every three hours. The second day I called on her and she could talk as well as ever.

I should like, my brothers, for you to give it a test for more thorough proving, and report at our next session.

